# SiO_2_/Zn_0.4_Co_0.6_Fe_2_O_4_ aerogel: an efficient and reusable superparamagnetic adsorbent for oily water remediation

**DOI:** 10.1039/d3ra03570k

**Published:** 2023-08-04

**Authors:** Fagr A. Shehata, Amer S. El-Kalliny, Mohamed S. Attia, Tarek A. Gad-Allah

**Affiliations:** a Water Pollution Research Department, National Research Centre 33 El Buhouth St., Dokki 12622 Giza Egypt kalliny78@hotmail.com as.el-kalliny@nrc.sci.eg +201012431344; b Chemistry Department, Faculty of Science, Ain Shams University Abbassia 11566 Cairo Egypt

## Abstract

Magnetic SiO_2_/Zn_0.4_Co_0.6_Fe_2_O_4_ aerogels were successfully prepared by sol–gel method with two different drying steps: ambient pressure drying (APD) and freeze-drying (FD). The surface chemistry of silica was modified to be hydrophobic by oleic acid. The prepared materials were fully characterized, displaying superparamagnetic behavior with saturation magnetizations of 10.2 and 15.1 emu g^−1^, and contact angles of ∼130° and ∼140° for the materials prepared by the APD and FD methods, respectively, indicating the hydrophobic surfaces of the prepared materials. This hydrophobicity allows the efficient separation of oil. Specifically, as high as 1.7 and 2 g g^−1^ adsorption capacities were obtained when using APD-dried and FD-dried silica aerogels, respectively, suggesting the preference for the FD method. Additionally, magnetic recovery and reuse of the adsorbents were successfully implemented in an attempt to reduce the overall practical application costs. To sum up, the prepared materials are good candidates for oil removal from wastewater and the protection of the environment.

## Abstract 2

Magnetic SiO_2_/Zn_0.4_Co_0.6_Fe_2_O_4_ aerogels were successfully prepared by sol–gel method with two different drying steps: ambient pressure drying (APD) and freeze-drying (FD).

## Graphical abstract

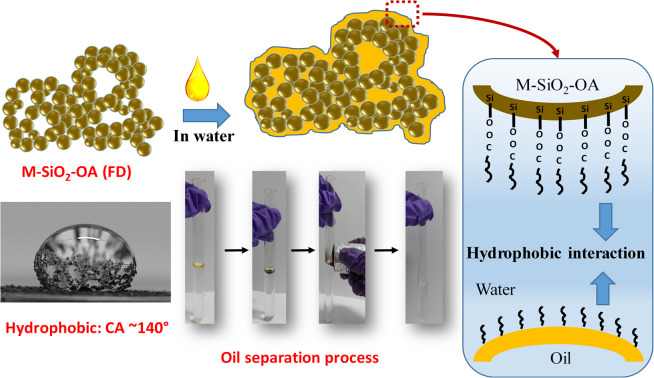

## 1 Introduction

Toxic compounds such as phenols, petroleum hydrocarbons, and polyaromatic hydrocarbons can be found in oily wastewater. They are known to be inhibitors of plant and animal growth.^1^ Thus, oil removal is a critical issue to avoid environmental problems. One of the promising wastewater techniques for oil removal is adsorption onto aerogels.

Generally, aerogels can be obtained by the sol–gel method, which includes several steps, including gel formation, aging, and drying.^2,3^ The drying step is crucial to avoid the collapse of the pores and maintain good textural properties. Commonly, there are three drying methods utilized: ambient pressure drying (APD), freeze-drying (FD), and supercritical drying.^4,5^ The supercritical drying method is based on the removal of the solvent from the gel using a supercritical fluid under very high pressure and temperature. The use of extremely hard conditions, such as high temperature and pressure, makes this method high in energy consumption, expensive, and unsafe.^2,5^ With FD, the solvent in the wet gel is frozen and removed by sublimation at low pressure using a simple, relatively affordable, and eco-friendly technique. APD is also a safe, economical, and ecologically friendly method that does not require complicated equipment. APD has attracted attention recently because of its simplicity and ease of use. In APD, surface modification, such as hydrophobization of the wet gel, is used to control capillary tension. As a result, capillary tension forces on the surface of the wet gel are reduced, leading to limited pore collapse.^2^

Recently, silica aerogels have shown promising performance in many applications (*e.g.*, catalysis, thermal insulation, and drug-delivery systems) because of their excellent properties, including high surface area, high porosity, low thermal conductivity, and low density.^6–8^ Silica aerogels were modified to be hydrophobic. This allowed the utilization of hydrophobic silica aerogels as adsorbents for the treatment of oily wastewater.^6,9^ For instance, Sorour *et al.*^9^ synthesized hydrophobic silica from sodium silicate and hexamethyldisilazane (HMDS) to treat saline and non-saline oily wastewater and achieved an oil removal efficiency of up to 96%. In the same research track, hydrophobic silica was implemented for the adsorption of three different types of oils. The adsorption experiments showed that the aerogel had a very high adsorption capacity, reaching up to 15.1 g g^−1^.^10^

Furthermore, the incorporation of magnetic materials with silica aerogels allows the magnetic separation, recovery, and reuse of silica aerogels. This feature is crucial in the field of water treatment because it significantly reduces the overall treatment cost. Shah *et al.* (2021)^11^ reported in their article review some examples of silica-based magnetic aerogels, which were used for different applications. In the field of water treatment, aerogels were combined with magnetite; for instance, Hu *et al.* (2016)^12^ prepared a Fe_3_O_4_/silica composite aerogel for the removal of rhodamine B and oil. In the same context, ferrites were incorporated with silica because of the superparamagnetic properties of ferrites, which enable a fast collection of the magnetic adsorbent in the presence of an external magnetic field and good dispersion in its absence. For example, Carta *et al.* (2013)^13^ synthesized zinc ferrite nanoparticles dispersed in a highly porous SiO_2_ aerogel.

However, methods employing ferrites with silica aerogel for oil removal have not been investigated yet. Therefore, this work is devoted to the preparation of a hydrophobic magnetic ferrite/silica aerogel composite, with an emphasis on the effect of the drying method on the oil separation efficiency. The proposed composite is SiO_2_/Zn_0.4_Co_0.6_Fe_2_O_4_ superparamagnetic aerogel adsorbent modified with oleic acid, which, to the best of our knowledge, has not been used for the removal of oil yet. Zn_0.4_Co_0.6_Fe_2_O_4_ was selected as the magnetic substance due to its high saturation magnetization and superparamagnetic behavior. The substitution of Co^2+^ ions in CoFe_2_O_4_ with 40% Zn^2+^ ions leads to enhanced magnetic properties of the cobalt ferrite nanomaterials,^14^ whereas oleic acid serves as a good hydrophobic agent.^15,16^ The magnetic aerogel adsorbent performance toward oil removal was studied under different factors: pH, the magnetic aerogel adsorbent dose, and the initial concentration of the oil. Finally, the reusability of the magnetic adsorbent was also studied.

## 2 Experimental methods

### 2.1 Synthesis of Zn_0.4_Co_0.6_Fe_2_O_4_ superparamagnetic nanoparticles

Zn_0.4_Co_0.6_Fe_2_O_4_ nanoparticles were prepared according to the previously reported procedure,^14^ except that zinc acetate was used instead of zinc nitrate. In this method, 1.616 g of Fe(NO_3_)_3_·9H_2_O (Loba Chemie Pvt Ltd, 98%), 0.1756 g of Zn(CH_3_COO)_2_·2H_2_O (Loba Chemie Pvt Ltd, 98%), and 0.349 g of Co(NO_3_)_2_·6H_2_O (Sigma-Aldrich, ≥98%) were dissolved in 40 mL of double distilled water (DDW) using a magnetic stirrer. Then, 1.28 g of NaOH (Loba Chemie Pvt Ltd, >98%) was dissolved in 10 mL of DDW and added drop-wise to the above solution. After that, the mixture was transferred to a 100 mL Teflon-lined autoclave and heated at 160 °C for 12 h in an electric furnace. The nanoparticles were collected using a permanent neodymium magnet (TM-30 × 50-N magnet, magnets4you Co., Germany), washed three times with DDW to remove inorganic byproducts, and twice with ethanol to remove organic byproducts. Finally, the nanoparticles were dried in an oven at 60 °C for 12 h under vacuum conditions.

### 2.2 Preparation of SiO_2_/Zn_0.4_Co_0.6_Fe_2_O_4_ magnetic aerogel adsorbent by the FD method

**Fig. 1:**
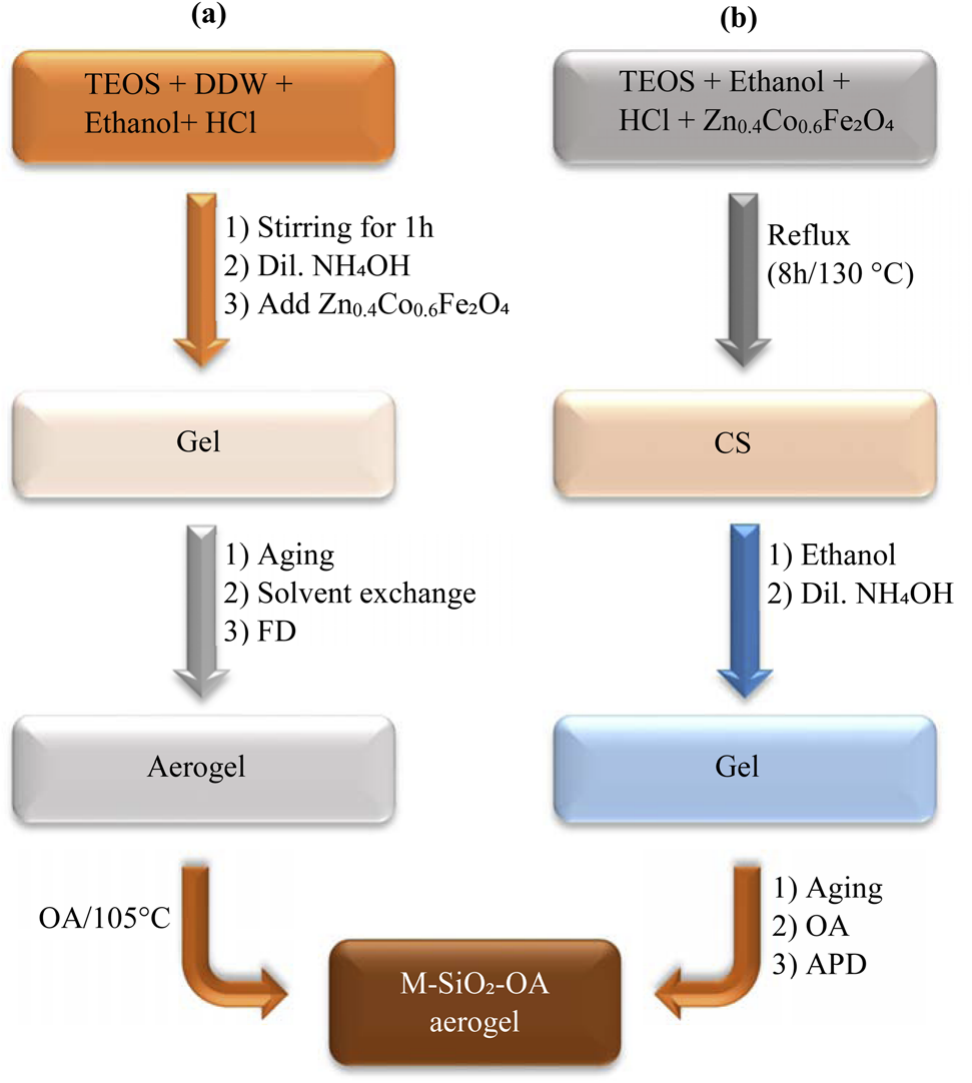
Experimental flowchart for the preparation of MSiO_2_-OA by (a) FD and (b) APD.

First, hydrophilic silica aerogel was prepared according to the reported procedure^17^, but with the addition of magnetic material. In this method (Fig. 1a), 8 mL of tetraethoxysilane (TEOS, Sigma-Aldrich, 98%), 4 mL of DDW, 19.2 mL of ethanol (Honeywell, ≥99.8%), and 0.32 mL of 1 wt% HCl solution (Acros Organics) were mixed for 1 h using an orbital shaker set at 250 rpm. After that, 1.7 mL of diluted ammonia (0.1 mol L^−1^) (Fisher Scientific Co.) was added to the mixture to form the alcosol. When the density of the sol increased, 1 g of Zn_0.4_Co_0.6_Fe_2_O_4_ was added to the mixture, resulting in a final ratio of 1 : 2 Zn_0.4_Co_0.6_Fe_2_O_4_ : SiO_2_. The mixture was shaken further until the gel formed. The prepared alcosols were kept in a closed beaker for one day for gelation at room temperature. After gelation, the gel was washed several times with ethanol. Then, the silica gel was aged at 50 °C for 3 h. After the solvent was exchanged with a mixture of DDW and isopropyl alcohol (Tedia), the wet gels were freeze-dried to obtain the hydrophilic magnetic aerogels (M-SiO_2_) powder.

For the surface modification, 1 g of the hydrophilic M-SiO_2_ was added to a mixture of 1.3 mL of oleic acid (OA) (Fluka Analytical Co.) and 11.7 mL of hexane (Fisher Scientific Co.). Then, the mixture was agitated in an ultrasonic bath at 50 °C until complete evaporation of hexane occurred. Subsequently, the residue was heated up to 105 °C (heating rate = 5 °C min^−1^) and maintained at this temperature for 1 h to ensure the occurrence of surface modification. The modified M-SiO_2_ was washed with hexane and ethanol several times to remove the unreacted oleic acid and byproducts and finally dried at 60 °C. This sample was designated as M-SiO_2_-OA (FD).

### 2.3 Preparation of SiO_2_/Zn_0.4_Co_0.6_Fe_2_O_4_ magnetic aerogel adsorbent by the APD method

Fig. 1b presents the experimental procedure followed for preparing the magnetic silica aerogel by the APD method. In this method, hydrophobic silica was prepared according to a reported procedure using ambient pressure drying (APD)^18^, but with the addition of magnetic material, and OA was used instead of TMCS as the hydrophobic agent. TEOS, ethanol, and diluted hydrochloric acid with the molar ratio of TEOS : ethanol : HCl = 1 : 4 : 1.2 (7.5 mL, 7.9 mL, and 1.23 mL, respectively) and 1 g of Zn_0.4_Co_0.6_Fe_2_O_4_ nanoparticles were added to obtain 1 : 2 ZnCoFe_2_O_4_ : SiO_2_ weight ratio. The whole mixture was refluxed at 130 °C for 8 h to allow the growth of condensed SiO_2_ (CS) in the presence of Zn_0.4_Co_0.6_Fe_2_O_4_. Then, 16.65 mL of the magnetic CS solution was diluted with 16.65 mL of ethanol. After that, 1.67 mL of ammonium hydroxide (28%) was added and shaken for 2 min. Then, the alcosol was aged at 80 °C for 2 h to allow the gelation. The grinding of the gel was done to produce a homogeneous fine powder.

Surface modification was done by adding the gel to a homogenous mixture of 18 mL of hexane and 2 mL of OA. The modified M-SiO_2_ was dried for 3 h at 105 °C (heating rate = 5 °C min^−1^) at ambient pressure. The dried sample was washed many times with hexane and then ethanol. Finally, the hydrophobic magnetic silica (M-SiO_2_-OA (APD) sample) was dried at 60 °C. For comparison, different samples of SiO_2_ were prepared either by FD or APD, but in the absence of Zn_0.4_Co_0.6_Fe_2_O_4_ and/or OA.

### 2.4 Characterization

Crystalline phases in the prepared materials were identified using X-ray diffraction (XRD) patterns, which were collected using a PANalytical X'Pert Pro diffractometer with a CuKα source (*λ* = 1.5406 Å). The magnetic properties were studied using a Riken Denshi BH-55 vibrating sample magnetometer (VSM) at room temperature. The functional groups of the prepared adsorbents were detected by Fourier transform infrared spectrometer (FTIR, Varian 3100 FT-IR Excalibur Series). The specific surface areas of the materials were calculated using the Brunauer–Emmett–Teller (BET) equation from the N_2_ adsorption–desorption isotherms collected using BELsorp Max (BEL Japan Inc.) surface analyzer. The morphological structures were examined by high-resolution transmission electron microscopy (HR-TEM, JEOL TEM-2100, Japan). The thermal properties were determined by thermogravimetric analysis (TG-DSC SETARAM Instrumentation Regulation, Labsys TM) at a heating rate of 15 °C min^−1^ in the air over the range of 40–1000 °C. To find out to what extent the prepared adsorbents were hydrophobic, contact angles were measured by the sessile drop method at room temperature. Briefly, a Plexiglas slide with a concave depression cavity was used and filled with the adsorbent samples; after that, the powder was pressed with another glass slide to form a uniform surface in order to prevent any water leakage. This configuration guaranteed that the water drop was only in contact with the surface of the powder. A water droplet was added to the surface of the adsorbent and a photograph was taken by the camera, and finally, the contact angle was measured using ImageJ software.

### 2.5 Adsorption performance

A vacuum pump oil (VPO) (Petronas vacuum pump oil ISO VG 68, Belgium) was used as the adsorbate in the adsorption experiments. The physical properties of the VPO are listed in Table 1. In these experiments, 10 mL of DDW and a certain amount of VPO were placed in a 20 mL test tube. Then, a certain amount of the hydrophobic adsorbent was added, and the mixture was mixed for 2 min using a vortex ((DAIHAN-Scientific “VM-10”). Then, the magnetic adsorbent with the adhered oil was separated using the permanent neodymium magnet. After that, 1 g of anhydrous sodium sulfate was dissolved in the oil–water mixture. Finally, the residual oil was extracted with 5 mL of hexane. The same steps were performed without adding any adsorbents to accurately measure the initial oil content. UV-visible spectrophotometer (JASCO V630, Japan) was used for measuring the residual oil content by recording the absorbance at 270 nm and a 9-point linear calibration curve with *R*^2^ = 0.999 in the range of 0.4 g L^−1^ to 20 g L^−1^.

**Table 1:** Physical properties of the VPO according to the manufacturer.

Parameters	Value
Density at 15 °C (g cm^−3^)	0.885
Viscosity at 40 °C (cp)	57.5
TAN (mg KOH g^−1^)	1.0

The adsorption capacity and the removal percentage of each adsorbent were calculated by the following equations:1
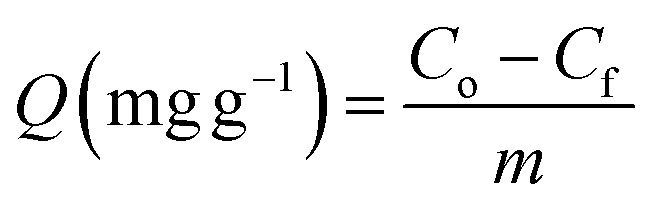
2
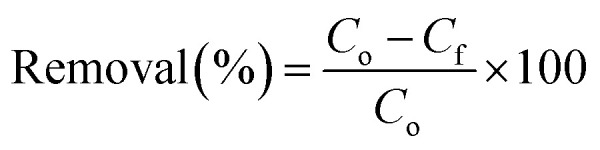
where, *Q* is the adsorption capacity, *C*_0_ and *C*_f_ are the initial and final concentrations (mg L^−1^) of oil, respectively, and *m* is the dose (g L^−1^) of the magnetic adsorbent used.

Different reaction conditions, including pH, the adsorbent dose, and the initial concentration of oil, were studied to optimize the adsorption process.

The reusability of the prepared adsorbents was also assessed. To do so, the magnetic adsorbents were collected by the permanent neodymium magnet and washed several times with hexane to remove the adherent oil, and then washed one time with ethanol. After that, the magnetic adsorbents were dried at 60 °C and then reused in the next cycle of oil adsorption.

## 3 Results and discussion

### 3.1 Characterization of the prepared materials

**Fig. 2:**
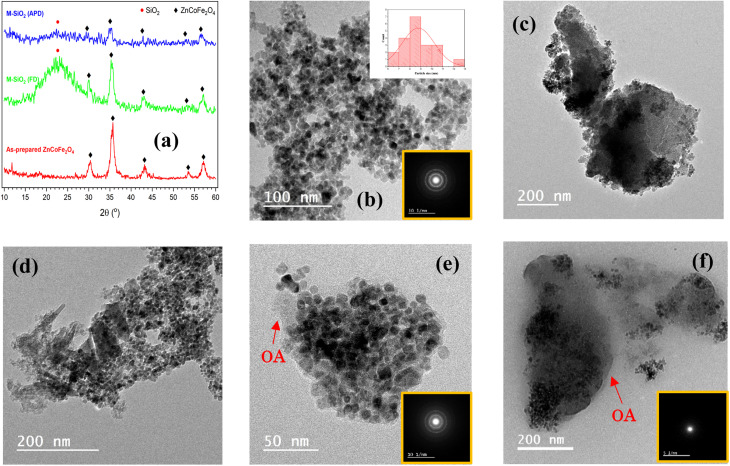
(a) XRD patterns of the prepared materials. HR-TEM images of (b) as-prepared Zn_0.4_Co_0.6_Fe_2_O_4_, (c) M-SiO_2_ (FD), (d) M-SiO_2_ (APD), (e) M-SiO_2_-OA (FD), and (f) M-SiO_2_-OA (APD). The SAED patterns are in the insets.

The X-ray diffraction patterns of the as-prepared Zn_0.4_Co_0.6_Fe_2_O_4_ superparamagnetic nanoparticles and the hydrophilic magnetic silica dried either by FD or APD are shown in Fig. 2a. In the as-prepared Zn_0.4_Co_0.6_Fe_2_O_4_ pattern, there were five diffraction peaks at 2*θ*° = 30.08°, 35.4°, 43°, 53.4°, and 56.9° corresponding to the (220), (311), (400), (422), and (511) diffraction planes of ZnFe_2_O_4_ and CoFe_2_O_4_ phases (ICDD #01-089-1009 and #00-022-1086, respectively). This indicates the formation of Zn_0.4_Co_0.6_Fe_2_O_4_ nanoparticles, according to Sunil *et al.*^14^ The same diffraction peaks of Zn_0.4_Co_0.6_Fe_2_O_4_ were recognized in both M-SiO_2_ (FD) and M-SiO_2_ (APD) patterns, with an additional broad peak between 20° and 30°, which corresponds to amorphous SiO_2_. Therefore, it can be deduced that the structure of Zn_0.4_Co_0.6_Fe_2_O_4_ was preserved in the composite samples. It is worth mentioning that all the diffraction peaks in the FD sample were of higher intensity than those in the APD sample. This might be due to the aggregation of SiO_2_ and Zn_0.4_Co_0.6_Fe_2_O_4_ as discrete grains in the M-SiO_2_ (FD) composite because Zn_0.4_Co_0.6_Fe_2_O_4_ was added to the SiO_2_ sol after partial gelation (*i.e.*, after increasing the viscosity of the SiO_2_ sol). Hence, the SiO_2_ network might be partially formed, leading to an inhomogeneous distribution of Zn_0.4_Co_0.6_Fe_2_O_4_ in the final composite. On the contrary, Zn_0.4_Co_0.6_Fe_2_O_4_ particles were suspended in the precursors' mixture to permit the condensation and gelation of SiO_2_ on Zn_0.4_Co_0.6_Fe_2_O_4_ surface in M-SiO_2_ (APD) sample. Thereby, the aggregation of SiO_2_ and Zn_0.4_Co_0.6_Fe_2_O_4_ as discrete particles was minimal in M-SiO_2_ (APD), leading to lower XRD peak intensities.

To confirm the above hypothesis, the morphologies of the prepared materials were imaged using HR-TEM, as shown in Fig. 2. The as-prepared Zn_0.4_Co_0.6_Fe_2_O_4_ appeared as well-defined cuboid particles ∼8.7 nm in average size (inset of Fig. 2b). The selected area electron diffraction (SAED) image (*i.e.*, inset of Fig. 2b) of these nanoparticles showed clear dotted rings, which could be attributed to the polycrystalline nature of this sample. Fig. 2c and d show the images of the M-SiO_2_ (FD) and M-SiO_2_ (APD) samples, respectively. In these samples, thicker sheets of silica and aggregates of Zn_0.4_Co_0.6_Fe_2_O_4_ were observed in the FD sample compared with the APD sample, which indicated the high intensity of the XRD peaks of the FD sample (see Fig. 2a). The image of the M-SiO_2_-OA (FD) sample in Fig. 2e shows that aggregates of Zn_0.4_Co_0.6_Fe_2_O_4_ were also obvious, leading to clear diffraction rings in the SAED image (inset of Fig. 2e) and high peak intensities in the relevant XRD pattern as presented above (see Fig. 2a). Also, the image shows a layer of OA surrounding the M-SiO_2_ (FD) particles, indicating the effective surface modification. Differently, the image of MSiO_2_-OA (APD) in Fig. 2f shows scattered Zn_0.4_Co_0.6_Fe_2_O_4_ nanoparticles within the amorphous SiO_2_ layer. In addition, a thin layer of OA was also noticeable. As a result, the SAED pattern of this material showed an amorphous nature. This is consistent with the XRD data shown in Fig. 2a.

**Fig. 3:**
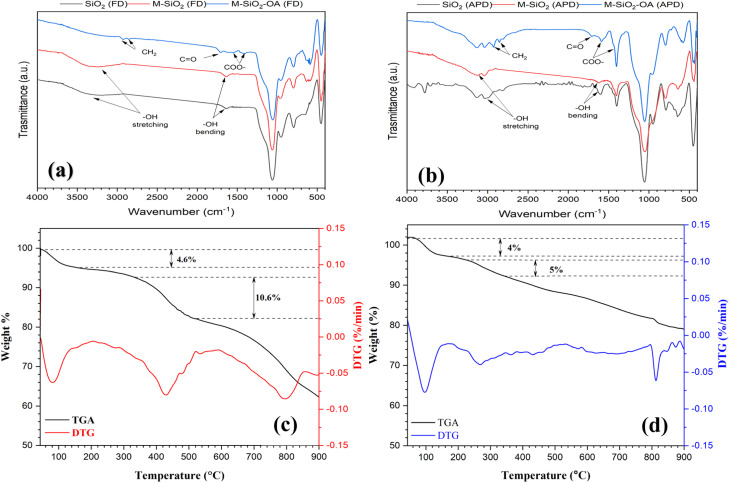
FTIR spectra of the prepared materials using (a) FD, and (b) APD methods. TGA- DTG curves of (c) M-SiO_2_-OA (FD), and (d) M-SiO_2_-OA (APD) samples.

The functional groups of the prepared materials were determined using FTIR spectroscopy, as illustrated in Fig. 3a&b. For SiO_2_ in Fig. 3a, the three peaks at 1058, 794, and 448 cm^−1^ could be attributed to the amorphous SiO_2_, which can be referred to –Si–O–Si– vibration modes.^19,20^ Additionally, a broad band in the range of 3688–2950 cm^−1^ and a small band at 1630 cm^−1^ were observed, which could be assigned to the stretching and bending vibrations of the O–H bonds.^21^ The same FTIR pattern was also noticeable in the M-SiO_2_ (FD) sample. After the surface modification with OA (*i.e.*, M-SiO_2_-OA (FD)), the peaks of the O–H bonds disappeared concurrently with the appearance of two new peaks at 1541 and 1406 cm^−1^, which are characteristics of the COO– group, and another small peak at 1708 cm^−1^, representing the C=O bond (Fig. 3a). This confirmed the formation of an ester bond between the silanol group and the carboxylic group of the oleic acid, *i.e.*, the chemical bonding between M-SiO_2_ and OA. Furthermore, the absorption peaks at 2928 and 2853 cm^−1^ were characteristic of the stretching vibrations of the –CH_2_ group,^22,23^ which further confirmed the presence of oleic acid in M-SiO_2_-OA (FD). The same trend was observed for the samples prepared using APD, as presented in Fig. 3b. In the case of SiO_2_ and M-SiO_2_ prepared by the APD method, there were also two small peaks at 1541 and 1406 cm^−1^, which are characteristics of the COO– group. These might be due to the residue from the precursors used in the APD preparation method. However, in the case of M-SiO_2_-OA (APD), the same peaks appeared with higher intensity, indicating the formation of an ester bond between the silica and the OA. Decisively, the FTIR data showed that the M-SiO_2_ particles had been successfully functionalized with oleic acid.

To quantitatively determine the OA amount in the composites, thermogravimetric analysis (TGA) and its differential form (DTG) were used. Irrespective of the peak at 80 °C arising from the loss of water, the DTG curve of MSiO_2_-OA (FD) showed a peak between 335.6 °C and 519.8 °C, corresponding to 10.6% weight loss in the TGA curve, as shown in Fig. 3c. In the case of M-SiO_2_-OA (APD) (Fig. 3d), only 5% weight loss was observed in the TGA curve, which appeared as a peak between 226.8 °C and 341 °C in the DTG curve. Given that the weight loss between 220 °C and 500 °C referred to the decomposition of OA according to ref. 24 and 25, the appearance of a peak in this range in both samples confirmed the formation of a hydrophobic surface. Furthermore, the TGA traces revealed that the amount of oleic acid on the surface of M-SiO_2_ (FD) was double that on the surface of M-SiO_2_ (APD), suggesting a better efficiency of M-SiO_2_ (FD) for oil–water separation.

**Fig. 4:**
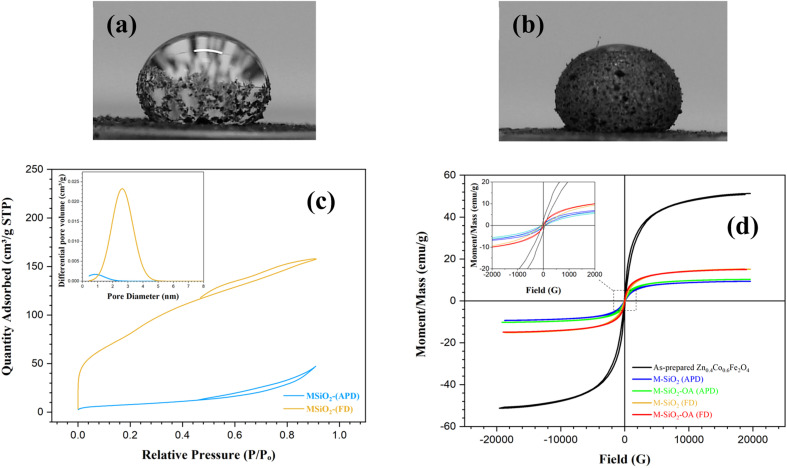
Contact angle measurements for (a) M-SiO_2_-OA (FD), and (b) M-SiO_2_-OA (APD). (c) N_2_ adsorption–desorption isotherms of the prepared materials with their pore-size distribution in the inset. (d) VSM hysteresis loops of the prepared samples.

The wetting properties of the prepared materials were assessed for further confirmation of the surface modification by OA. This was performed by measuring the contact angles after adding a water drop to the surface of the samples. For the unmodified samples (*i.e.*, M-SiO_2_ (FD) and M-SiO_2_ (APD)), the water droplet penetrated rapidly into the surface of the sample in less than a second. So that, the contact angle could not be measured, indicating the hydrophilicity of the surface. After surface modification, the contact angles of M-SiO_2_-OA (FD) and M-SiO_2_-OA (APD) were ∼140° and ∼130°, respectively, as shown in Fig. 4a&b. This points to the hydrophobic surface of both materials. However, the sample prepared with the FD method showed more hydrophobicity than that prepared by the APD method because of the higher OA content, as evidenced by the TGA data (see Fig. 3c&d).

More information about the textural characteristics of the hydrophilic materials might explain the differences in their tendencies toward surface modification. Thereby, the textural characteristics were assessed based on the collected N_2_ adsorption–desorption isotherms presented in Fig. 4c. M-SiO_2_ (FD) showed a Type II isotherm according to the IUPAC classification.^26^ This type characterizes physical adsorption on nonporous or macroporous adsorbents with uncontrolled monolayer–multilayer adsorption up to a high *P*/*P*_o_. On the contrary, the M-SiO_2_ (APD) sample exhibited a Type III isotherm, revealing a relatively weak interaction between the adsorbent and adsorbate on the surface of nonporous or macroporous substances. In this case, the adsorbed molecules aggregate around the most suitable sites.^26^ Therefore, in the modification step, OA might be adsorbed on the whole surface of M-SiO_2_ (FD), while it only aggregated at specific locations on the M-SiO_2_ (APD) surface. Accordingly, surface modification with OA was more pronounced in the case of M-SiO_2_ (FD) than M-SiO_2_ (APD). This speculation is consistent with the contact angle and TGA results shown above.

The higher OA content in M-SiO_2_ (FD) could also be attributed to the BET-specific surface area (*S*_BET_) and the total pore volume, which were 325 m^2^ g^−1^ and 0.25 cm^3^ g^−1^, respectively, *versus* only 30 m^2^ g^−1^ and 0.08 cm^3^ g^−1^ for M-SiO_2_ (APD). Therefore, the sample prepared using FD had a ten-fold higher surface area available for OA adsorption than the sample dried with APD. Additionally, the wider pores in M-SiO_2_ (FD) were more accessible for OA than those in M-SiO_2_ (APD), as observed in the TEM results. Accordingly, most of the OA was localized in the pores of the FD sample, while it was dispersed on the APD sample surface. To sum up, the textural properties of M-SiO_2_ (FD) were more suitable for surface modification with OA than those of M-SiO_2_ (APD). The surface area of the modified hydrophobic adsorbents could not be measured as a layer of oleic acid covered the surface and partially occupied the pores of M-SiO_2_, which was confirmed by the TEM data (see Fig. 2e&f).

The magnetic properties are important to assess the possibility of magnetic separation and recovery of the prepared materials. These magnetic properties were extracted from the VSM loops presented in Fig. 4d. The as-prepared Zn_0.4_Co_0.6_Fe_2_O_4_ clearly showed the hysteresis loop characteristic of superparamagnetic materials. Interestingly, this superparamagnetic character was not altered even after adding the adsorbent and the hydrophobic agent (*i.e.*, OA), *i.e.*, the prepared materials had nearly zero coercivity and zero retentivity, confirming the superparamagnetic behavior. The measured saturation magnetizations (*M*_s_) of Zn_0.4_Co_0.6_Fe_2_O_4_, M-SiO_2_ (FD), M-SiO_2_-OA (FD), M-SiO_2_ (APD), and M-SiO_2_-OA (APD) were 51, 15.2, 15.1, 9.3, and 10.2 emu g^−1^, respectively. As expected, the *M*_s_ value of Zn_0.4_Co_0.6_Fe_2_O_4_ decreased after the addition of the non-magnetic SiO_2_ aerogel. However, insignificant changes in *M*_s_ values were recorded in the hydrophilic and hydrophobic samples, which might be due to the low content of OA in the hydrophobic samples. Surprisingly, the samples prepared using APD samples showed lower *M*_s_ values, which could be attributed to the scattering of Zn_0.4_Co_0.6_Fe_2_O_4_ particles within SiO_2_. However, all the samples still possessed the magnetic character necessary for their recovery after they applied for oil removal.

### 3.2 Performance of the adsorbents

**Fig. 5:**
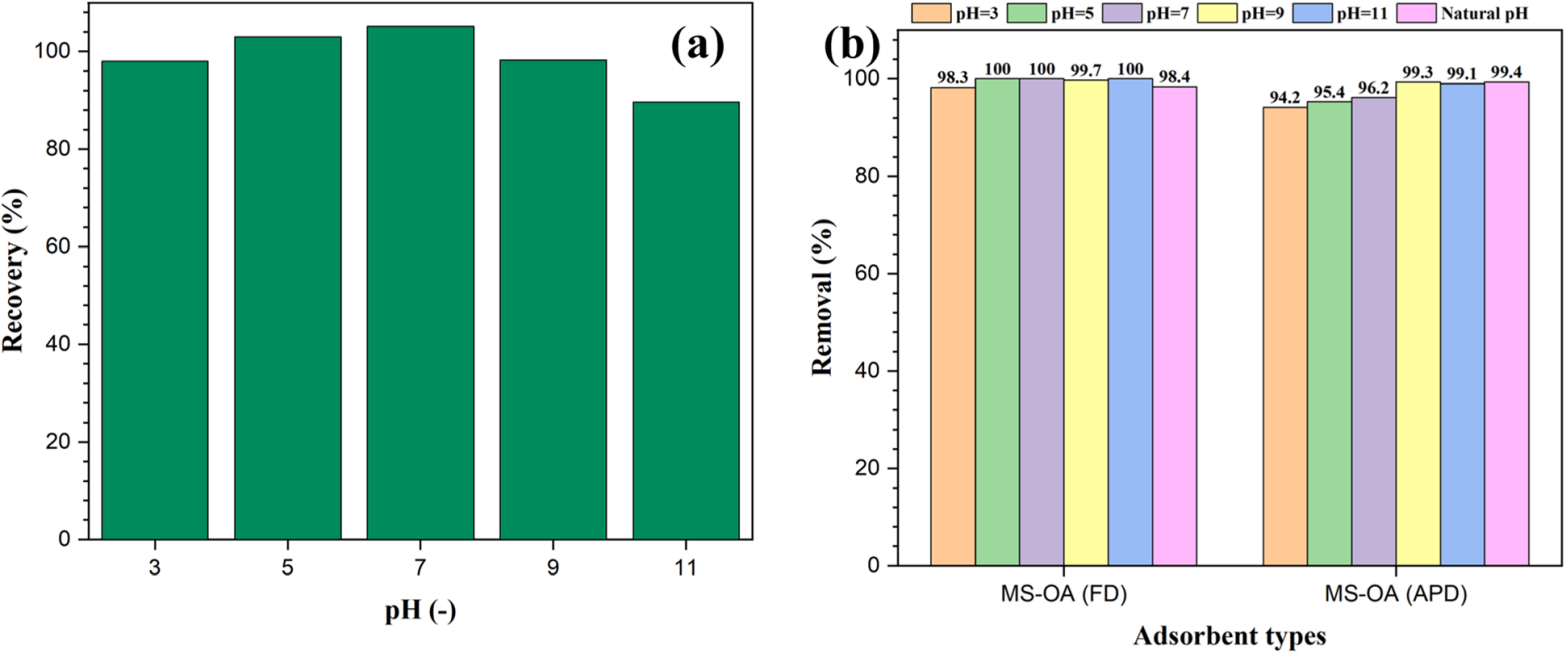
Effect of pH on (a) oil recovery in the absence of the magnetic adsorbent, and (b) oil removal by the magnetic adsorbents.

The ability of the hydrophobic adsorbents for oil removal was determined by measuring the absorbance of the residual oil after adding the prepared materials to an oil–water immiscible mixture. Different reaction conditions were investigated to optimize this process. For instance, the pH value of the medium was changed in the range of 3–11 using either 0.1 M HCl or 0.1 M NaOH to study the effect of pH on oil removal. First, the recovery of VPO in the absence of the magnetic adsorbent at different pH values was investigated to avoid any misleading data due to the effect of pH on the analysis of the residual oil. According to the data in Fig. 5a, the recovery percentage ranged between 90% and 105%, indicating successful oil recovery within the studied pH range. To determine the optimum pH for oil adsorption, 50 mg of the magnetic adsorbent was added to a test tube containing a mixture of 50 mg of VPO in 10 mL of water adjusted at different pH values. For both the M-SiO_2_-OA (FD) and M-SiO_2_-OA (APD) samples, very high removal efficiencies were recorded under the studied pH range with no significant variation (Fig. 5b). This supports the applicability of the prepared materials over a wide pH range. The natural pH was then selected to perform further investigation.

**Fig. 6:**
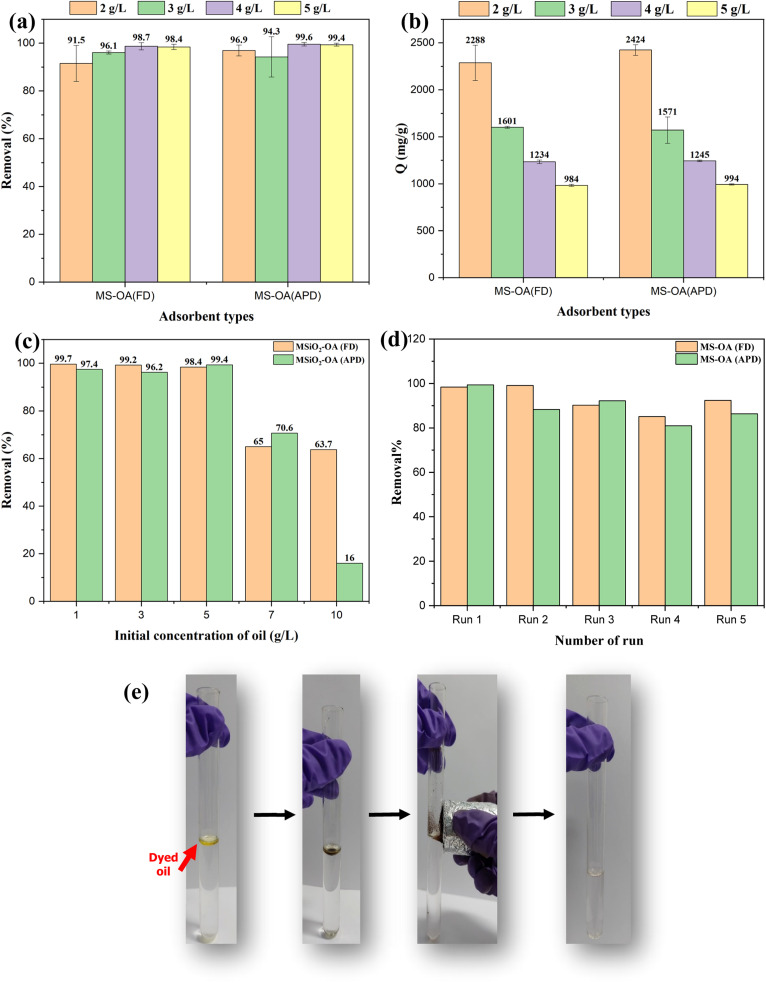
Oil removal by the prepared materials under different conditions; (a and b) effect of the adsorbent dose on the oil removal and adsorption capacity, respectively, (c) effect of the initial concentration, (d) reusability test, and (e) Photographic image of the oil separation process.

Fig. 6a shows the effect of the adsorbent dose at a natural pH and with an initial oil concentration of 5 g L^−1^ for both types of adsorbents. The oil–water separation performance was independent of the adsorbent dose and reached a very high value in the range of 91.5–99.6% for all the studied concentrations. The calculated maximum adsorption capacities were 2.3 g g^−1^ and 2.4 g g^−1^ for M-SiO_2_-OA (FD) and M-SiO_2_-OA (APD), respectively, as shown in Fig. 6b. The obtained adsorption capacities are comparable with those reported in the literature when using oils of similar viscosity to the VPO used in this study. It is important to mention that the adsorption capacity of magnetic adsorbents depends not only on the nature of the adsorbent but also on the physical properties of the adsorbate (*i.e.*, oil). Low-viscosity oil could diffuse through the porous surfaces of the adsorbents, increasing their adsorption capacity. However, high-viscosity oil adheres on the surface of the adsorbent without penetration into the internal pores, leading to a low adsorption capacity.^27–29^ For example, Yu *et al.* (2020)^30^ prepared poly (tripropylene glycol diacrylate) (PTPGDA) magnetic porous microspheres for oil removal with adsorption capacities of 2.58, 1.46, 2.36, 3.22, and 2.24 g g^−1^ for diesel, soybean oil, kerosene, xylene, and EGDMA, respectively. In their study, the adsorption capacity for soybean oil, which has the highest viscosity (50 cp), was small compared with the other adsorbates. In addition, Rabbani *et al.* (2021)^31^ synthesized Carbonyl iron @ glucose@ stearic acid for oil–water separation with adsorption capacities of 4.1, 2.5, 3.1, and 3.7 g g^−1^ for hexane, silicone oil, gasoline, and kerosene, showing a low adsorption capacity for the viscous silicone oil. To sum up, the nature of the adsorbate plays a role in the adsorption mechanism, which affects the adsorption capacity of the adsorbent.

To study the effect of the initial VPO concentration, 2 g L^−1^ of the adsorbents was used at a natural pH, and the concentration of oil was varied from 1 g L^−1^ to 10 g L^−1^, as shown in Fig. 6c. At low oil concentrations, the efficiency of oil removal almost reached ∼100%. When the initial concentration was increased, the oil removal efficiency decreased, as expected. Comparing the adsorbents at high initial concentrations revealed that M-SiO_2_-OA (FD) showed not only high efficiency for oil removal but also better stable performance even at high oil concentrations. This might be due to the higher OA content in M-SiO_2_-OA (FD), as evidenced by the characterization of the prepared materials.

### 3.3 Reusability test of the adsorbents

The ultimate goal of having a magnetic adsorbent is the successful separation and reuse of this adsorbent. Consequently, 5 g L^−1^ of both hydrophobic adsorbents was reused for oil/water separation (5 g L^−1^ of initial VPO concentration) five times at natural pH. The collected results and photographic images for the oil separation process are presented in Fig. 6d&e. For the OA-modified particles, it was found that there was no significant change in the efficiency of oil removal even after five runs, demonstrating the stability of the adsorbents as the hydrophobicity of the adsorbent did not change after several cycles. After the 5th run, the efficiency of oil removal decreased by ∼6% and ∼13% for M-SiO_2_-OA (FD) and M-SiO_2_-OA (APD), respectively. These results recommend the applicability of M-SiO_2_-OA (FD) because it demonstrated effective oil removal and better reusability than M-SiO_2_-OA (APD).

### 3.4 Proposed adsorption mechanism

**Fig. 7:**
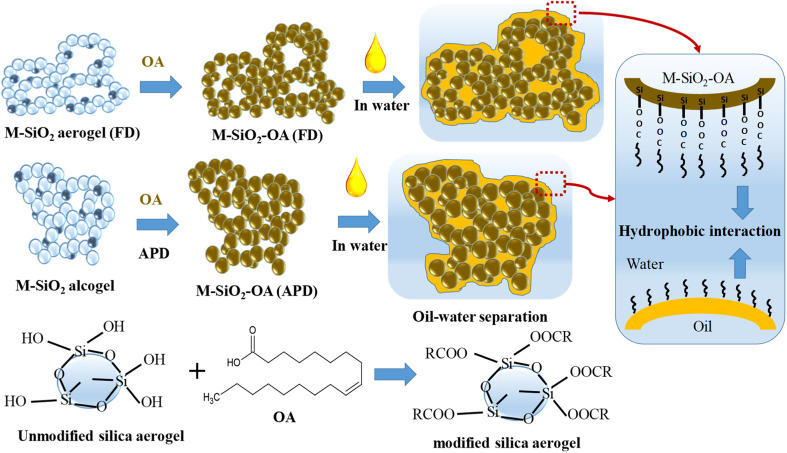
The proposed oil–water separation mechanism.

Fig. 7 depicts the proposed mechanism of oil separation by the prepared magnetic aerogels. The surface modification of M-SiO_2_ by OA was carried out through the chemical binding of the OH groups on the surface of the silica particles with the carboxylic groups in OA to form ester groups (see FTIR data in Section 3.1). This chemical binding explains the stability of the prepared M-SiO_2_-OA adsorbents, which was reflected in the good reusability performance (see reusability data in Section 3.3). OA covers the surface of the prepared M-SiO_2_ aerogels and partially occupies their pores (see BET data in Section 3.1) converting them to be selective adsorbents for oil removal by introducing a hydrophobicity character. In the oil–water separation process, as presented in Fig. 7, the physisorption of the oil occurs by hydrophobic–hydrophobic interactions with the OA over the surface of the modified M-SiO_2_ aerogels. The same behavior was observed by Huang and Yan (2018) with their suggested pore-filling mechanism for oil–water separation using a resilient graphene aerogel.^32^ Furthermore, the amount of oleic acid on the surface of M-SiO_2_ (FD) was double that on the surface of M-SiO_2_ (APD) (see TGA data in Section 3.1), giving rise to the better performance of the M-SiO_2_-OA (FD) adsorbent.

## 4 Conclusion

In this work, oleic acid-modified magnetic silica aerogels were successfully prepared using two different drying methods: ambient pressure drying (APD) and freeze-drying (FD). The prepared M-SiO_2_-OA (APD) and M-SiO_2_-OA (FD) showed superparamagnetic behaviors with saturation magnetizations of 10.2 and 15.1 emu g^−1^, and contact angles of ∼130° and ∼140°, respectively, indicating more *M*_s_ and a greater hydrophobicity of the materials prepared by the FD method. Practically, this superparamagnetic character enabled the fast collection of the magnetic adsorbent in the presence of an external magnetic field and good dispersion in its absence. The surfaces of the materials were chemically modified by OA, as evidenced by the FTIR analysis. Chemical binding gives high stability to the prepared magnetic adsorbents, which was reflected on their good reusability performance. M-SiO_2_-OA (FD) was demonstrated to be more efficient for oil removal, even at high oil concentrations. Physisorption can describe the oil–water separation process *via* hydrophobic–hydrophobic interactions with the OA over the surface of the modified M-SiO_2_ aerogels. Moreover, it was found that M-SiO_2_-OA (FD) could be reused for up to 5 cycles using magnetic separation without a significant loss of removal efficiency and with higher stability compared with M-SiO_2_-OA (APD). Therefore, the M-SiO_2_-OA (FD) sample can be considered a highly efficient and reusable adsorbent for the removal of oil spills, which can be applied practically.

## Conflicts of interest

There are no conflicts to declare.

## Supplementary Material

## References

[cit1] Yue X., Li Z., Zhang T., Yang D., Qiu F. (2019). Chem. Eng. J..

[cit2] Ebrahimi A., Dahrazma B., Adelifard M. (2020). J. Porous Mater..

[cit3] Goryunova K., Gahramanli Y., Gurbanova R. (2023). RSC Adv..

[cit4] Lee J.-H., Park S.-J. (2020). Carbon.

[cit5] Kaya G. G., Aznar E., Deveci H., Martinez-Manez R. (2021). Biomater. Sci..

[cit6] Sert Çok S., Koç F., Gi̇zli̇ N. (2021). J. Hazard. Mater..

[cit7] Lu J., Wang J., Hassan K. T., Talmantaite A., Xiao Z., Hunt M. R. C., Šiller L. (2020). Sci. Rep..

[cit8] Zhao C., Li Y., Ye W., Shen X., Yuan X., Ma C., Cao Y. (2021). J. Non-Cryst. Solids.

[cit9] Sorour M. H., Hani H. A., Al-Bazedi G. A., El-Rafei A. M. (2016). J. Porous Mater..

[cit10] Wang D., McLaughlin E., Pfeffer R., Lin Y. S. (2012). Sep. Purif. Technol..

[cit11] Shah N., Rehan T., Li X., Tetik H., Yang G., Zhao K., Lin D. (2021). RSC Adv..

[cit12] Hu S.-C., Shi F., Liu J.-X., Yu L., Liu S.-H. (2016). J. Porous Mater..

[cit13] Carta D., Marras C., Loche D., Mountjoy G., Ahmed S. I., Corrias A. (2013). J. Chem. Phys..

[cit14] Sunil K. C., Utsav S., Nairy R. K., Chethan G., Shenoy S. P., Mustak M. S., Yerol N. (2021). Ceram. Int..

[cit15] Liang J., Du N., Song S., Hou W. (2015). Colloids Surf., A.

[cit16] Yang Y., Cheng T., Wu H., You Z., Shang D., Hou J. (2020). Energy Fuels.

[cit17] Sai H., Xing L., Xiang J., Cui L., Jiao J., Zhao C., Li Z., Li F. (2013). J. Mater. Chem. A.

[cit18] Liu R., Wang J., Du Y., Liao J., Zhang X. (2019). J. Solid State Chem..

[cit19] Kurnaz Yetim N., Kurşun Baysak F., Koç M. M., Nartop D. (2020). J. Mater. Sci.: Mater. Electron..

[cit20] Fan Q., Guan Y., Zhang Z., Xu G., Yang Y., Guo C. (2019). Chem. Phys. Lett..

[cit21] Premaratne W., Priyadarshana W., Gunawardena S., De Alwis A. (2013). J. Sci. Univ. Kelaniya.

[cit22] Ibarra J., Melendres J., Almada M., Boa M. G., Taboada P., Juárez J., Valdez M. A. (2015). Mater. Res. Express.

[cit23] Prévot G., Mornet S., Lorenzato C., Kauss T., Adumeau L., Gaubert A., Baillet J., Barthélémy P., Clofent-Sanchez G., Crauste-Manciet S. (2017). Data Brief.

[cit24] Kukreja A., Kang B., Han S., Shin M.-K., Son H. Y., Choi Y., Lim E.-K., Huh Y.-M., Haam S. (2020). Nano Convergence.

[cit25] Rimal V. , Shishodia S. and Srivastava P., Novel synthesis of high thermal stability carbon dots & nanocomposites from Oleic acid as an organic substrate, 2019

[cit26] Thommes M., Kaneko K., Neimark A., Olivier J., Rodriguez-Reinoso F., Rouquerol J., Sing K. (2015). Pure Appl. Chem..

[cit27] Liu R.-L., Li X.-Q., Liu H.-Q., Luo Z.-M., Ma J., Zhang Z.-Q., Fu Q. (2016). RSC Adv..

[cit28] Wong L. Y., Lau S. Y., Pan S., Lam M. K. (2022). Chemosphere.

[cit29] Xu G., Zhang L., Yu W., Sun Z., Guan J., Zhang J., Lin J., Zhou J., Fan J., Murugadoss V. (2020). Nanotechnology.

[cit30] Yu Y. L., Zhou Z. H., Yu W. T., Song Y. Z., Xie M. Q. (2020). Macromol. Mater. Eng..

[cit31] Rabbani Y., Shariaty-Niassar M., Ebrahimi S. A. S. (2021). Ceram. Int..

[cit32] Huang J., Yan Z. (2018). Langmuir.

